# Porcine Cysticercosis Control in Western Kenya: The Interlink of Management Practices in Pig Farms and Meat Inspection Practice at Slaughter Slabs

**DOI:** 10.1155/2020/7935656

**Published:** 2020-08-27

**Authors:** Marie-Françoise Mwabonimana, Charles Muleke Inyagwa, Bockline Omedo Bebe, Eduard Kokan Shakala, Anthony Macharia King'ori

**Affiliations:** ^1^Department of Animal Science, Faculty of Agriculture, Egerton University, P.O. Box 536–20115, Egerton, Kenya; ^2^College of Animal Sciences and Veterinary Medicine, University of Rwanda, P.O. Box 210, Musanze, Rwanda; ^3^Department of Veterinary Medicine, Faculty of Veterinary Medicine and Surgery, Egerton University, P.O. Box 536-20115, Egerton, Kenya

## Abstract

This study assessed the management practices for controlling porcine cysticercosis (PC) on pig farms and in pork at the slaughter slabs in two counties (Busia and Kakamega) of Western Kenya. A total of 162 pig-rearing households at the farm level, 26 butcher owners, and 26 slaughter slab workers at the slaughter slab level were interviewed using a structured questionnaire. Data were analyzed using the “Statistical Analysis System” (SAS) programme. Results indicated that the frequent management practices used at the farm level (*p* < 0.05) were rearing pigs under free range (69.1%), latrine ownership by households (87.7%), and use of pit latrines (72.8%) in households. At the slaughter level (*p* < 0.05), results of the butcher owners (76.9%) and slaughter slab workers (62.5%) revealed that meat inspection was not practiced adequately in the two areas of study. The results imply that slaughtered pigs for human consumption were not adequately inspected, and thus, the study recommends for implementation of effective pig management practices at the farm level and pork meat inspection at slaughter slabs to prevent PC infections and assure food safety along the pork value chain.

## 1. Introduction

Porcine cysticercosis (PC) is an infection of pigs which is prevalent in many developing countries [1] with high effect on public health and agriculture [2, 3]. The disease is caused by *Taenia solium* which also causes cysticercosis in pigs, seizures and death in pigs [4, 5], and epilepsy in humans [6, 7]. The zoonotic tapeworm *T. solium* has a two-host life cycle: the indirect one in humans as the definitive host harboring the mature tapeworm in the small intestine, causing taeniasis and the second with pigs as a normal intermediate host harboring the larval *Cysticerci* which encyst in the muscles and brain and cause porcine cysticercosis [8]. Transmission of *T. solium* is related to socioeconomic, behavioural, and environmental factors [9, 10]. This was confirmed in a study in Western Kenya [11] which reported that inadequacy in meat inspection, sanitation, and cooking habits were contributing factors to cysticercosis transmission for *Taenia* spp. Contact with infected human faecal waste by pigs is a requisite for the successful propagation of the parasite's lifecycle [12].

In pig farming, external and internal biosecurity measures are critical tools in preventing the transmission of diseases, contributing to public health and improving livelihood of pig farmers [13]. Biosecurity encompasses bioexclusion, biocontainment, and biomanagement. The three practices are distinct but often blended with sets of actions and overlapping components. Most often, pig producers focus on bioexclusion and biomanagement while neglecting biocontainment which is the prevention of the spread of disease agents to neighbors or even long-distance transfer. In bioexclusion, the external biosecurity involves preventing the introduction of new pathogens/diseases within a pig unit from outside source, while biomanagement refers to the combined effort to control economically important infectious diseases that are already present in the farm population [14].

The observation of routine farm biosecurity constitutes a priority solution in the minimization of risk in disease spread [15]. It has been documented that total confinement of pigs poses welfare issues and could create other management problems such as aggressiveness and biting [16–18]. The feasibility of the intensification of livestock production requires long-term application of the One Health approach [19], focusing on the mitigation of the health risks at the interfaces between animals and humans in different ecosystems [20]. Studies elsewhere have reported that safe slaughter of pigs and monitoring of rejected carcasses found to be infected at the farm level contributed to the interruption of the parasite life cycle [21]. Poor implementation of biosecurity measures exposes pigs to the risk of PC disease [18, 22]. Estimating the extent of the risks of PC and its consequences to pig farming requires well-maintained and updated pig production and management records. However, the veterinary reports, farm records, and other important statistics on pig farming are usually absent, inaccurate, or completely missing in various households and slaughter slabs. This study was undertaken to determine the management practices frequently used by pig-rearing farmers and the level of implementation of meat inspection at various slaughter slabs in Busia and Kakamega counties of Western Kenya.

## 2. Materials and Methods

### 2.1. Study Site and Questionnaire

**Figure 1:**
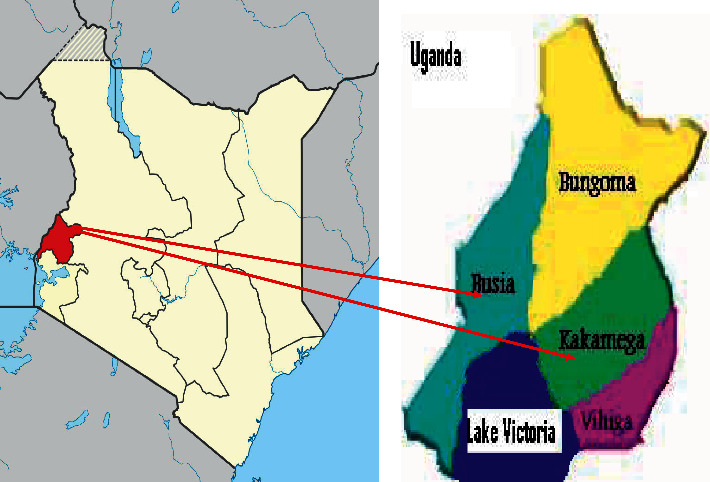
Map of Kenya showing Western Kenya (red color). The red arrows show Busia and Kakamega counties. Source: Kenya National Bureau of Statistics, 2019.

This study was conducted from August to September 2018 within 9 villages within Busia and Kakamega Counties (Figure 1), two of the forty‐seven (47) Counties of Kenya situated in the Western Kenya. The Western Region is located in the Western part of Kenya and borders of Uganda. It covers an area of 8,361 km2 (2,867.3 sq miles) and an estimated population of 5,021,843 (Census 2019) and population density of 590/km2 (1,500/sq mi). The climate is mainly tropical, with variations by County due to altitude. The whole region experiences heaviest rainfall in April and lowest in January, with the long rains which is at its peak between late March and late May. The minimum temperatures range from 14°C to 18°C and maximum of 30°C to 36°C throughout the year (24). The villages have high concentration of free scavenging pigs within Busia (Mundika, Bugengi, Nango'ma, Lwanya, Murende) and Kakamega Counties (Shikulu, Shivagala and Lunenele for Idako central), Mukongolo for Idakho North). The human population at risk of taeniasis of Busia and Kakamega is 893,681 and 1,867,579, respectively [23].

Qualitative data on management practices influencing the disease were collected through interview using structured questionnaires which were translated in the national language and local language for some respondents during the interview. A structured questionnaire on pig farming management practices at the farm level was administered to 162 pig-rearing smallholder households, on the prevailing management practices. The pig-rearing smallholders were composed by 102 (63.75%) from Busia and 60 (36.25%) from Kakamega, respectively. A separate questionnaire on meat inspection implementation at the slaughter slab level was administered to 26 licensed butcher owners who brought their pigs at the slaughter slabs during the period of the data collection and 26 slaughter slab workers to collect information on the level of implementation of meat inspection. All slaughter slabs (Khayega, Shinyalu, and Malinya from Kakamega county; Musambaruwa and Matayos from Busia county) in the selected clusters were sampled. Variables defining management practices and meat inspection implementation were collected using the binary response [24] from farmers and slaughter slabs. Respondents would indicate whether they had frequently (yes) or had not frequently practiced (no) against a set of nine measures of management practices, namely, free-range pig keeping, use of outdoor defecation by humans, presence of latrine by the household, using of pit latrines by the household, sourcing water outside the farm, sourcing feed outside the farm, routine deworming, routine vaccination, presence of a fenced farm, and meat inspection (Table 1).

### 2.2. Data Analysis

Qualitative data on management practices from pig-rearing households, butcher owners who approached at the slaughter slabs. and slaughter slab workers were entered into Microsoft Excel (2007) and exported to SAS version 9.1.3 [24] for analysis. Descriptive statistics were used to summarize respondents' demographic characteristics and management practices [26].

## 3. Results

### 3.1. Demographic Characteristics of Farmers and Butcher Owners by Counties

A total of 214 respondents comprising 162 pig-rearing households, 26 butcher owners, and 26 slaughter slab workers were interviewed at the farm and slaughter slab points in Busia and Kakamega counties of Western Kenya. Out of the 162 pig-rearing households interviewed, majority, 37.7%, 26.5%, and 10.5% were youthful farmers whose age groups varied between 21 and 30, 31 and 40, and 11 and20, respectively. One-quarter (25.5%) of the households interviewed were over 41–50 years old, and 53.1% belonged to the female gender, while 41.7% had no formal school education. A majority (77.2%) of farmers in Busia and Kakamega counties had kept pigs for a period of 6–10 years, while 22.8% had kept them for an average period of 28–35 years (Table 2).

For butcher owners, out of the 26 respondents interviewed, majority, 53.9% were between 11 and 20 years old, 92.3% of them were male gender, and 57.7% % had secondary school education. A majority (46.2%) of butcher owners in Busia and Kakamega counties had sold pigs for a period of 6–10 years (Table 2).

### 3.2. Management Practices Preventing PC Infection at the Production Level

The results (Table 3) indicate that, in the two counties (Busia and Kakamega), more farmers frequently practiced (*p* < 0.05) free-ranging pig rearing (69.1%), have latrines (87.6%), and used latrines (72.8%). However, more farmers did not frequently (*p* < 0.05) practice use of outdoor defecation (66.7%), vaccination (69.7%), routine deworming (70.4%), fencing the farm (77.8%), and sourcing water (92.0%) or sourcing feed (87.0%) outside the farm.

### 3.3. Management Practice Influencing PC Infection at the Slaughter Slabs

Results from Table 4 show the attitudes of butcher owners and slaughter slab workers towards the level of implementation of meat inspection as a management practice at slaughter slabs. However, more of the butcher owners (76.9%) and slaughter slab workers (61.5%) attested that the meat inspection is frequently (*p* < 0.05) practiced, and 23.1 and 38.1% of them, respectively, did not attest that.

## 4. Discussion

This paper describes the pig farming management practices and meat inspection implementation at farm and slaughter slab levels to investigate factors favouring porcine cysticercosis in Busia and Kakamega counties. The demographic descriptors revealed that out of the 162 farmer population interviewed, 37.7% were aged between 21 and 30 years, 53.1% were of the female gender, 41.4% had no formal school education, and 77.2% had kept pigs for a period of 6 to 10 years (Table 2). These findings were similar to those reported that the female gender dominated rearing and owning pigs in the rural areas of Western Kenya [26, 27] and other African countries [28–30]. The findings agree with the report by Ampaire and Totchschild [31] that, in Africa, women are traditionally empowered to rear and own pigs as opposed to cattle. These findings differed from early reports on pig farmer age ranges of 12–88 and 45–60 years in Homa Bay and Embu counties of Kenya [32–34]. They also reported that 86.4% and 92.6% pig farmers in Uganda and Kenya (Embu county), respectively, were males. This variation could be attributed to the sociocultural differences in the areas of this study.

Pigs in the two counties were predominantly reared under the free-range system at the farm level (69.1%) (Table 3). The presence of latrines at households and use of structurally dilapidated, unhygienic pit latrines for human waste disposal formed the main bioexclusion, biocontainment, and biomanagement practices with a frequency of up to 87.7% and 72.8% in the surveyed farms. Studies elsewhere had established a significant positive relationship between inappropriate use of latrines and PC prevalence [33, 35]. It has been documented that keeping pigs under the free-range system elevated the risk of pigs acquiring *T. solium* infection that leads to the endemicity of zoonotic porcine cysticercosis [36]. Findings in this study not only concurred with this fact but also corroborated the information that pigs kept under the free-range pig production system, compounded by poor utilization or lack of latrines, could have been the main contributing factors for the spread and endemicity of PC in the two counties at the farm level.

In this study, 76.9 and 61.5% of butcher owners and slaughter slab workers reported that meat inspection was frequently implemented at slaughter slabs (Table 4). It was observed that meat inspection practice was occasionally ignored in some slaughter slabs in seasons of high demand and was not thoroughly performed in the sense that infected animals could be slaughtered, and uninspected meat easily found its way into the human food chain. The observations here concur with those given by Gabriël et al. [37], who reported that inadequate meat inspection was a contributory factor to the spread of the infection by *Taenia solium* which could lead to the emergence or re-emergence of the disease in pig farming systems. This finding suggests that inadequate meat inspection at the slaughter slabs is a critical factor influencing the spread of this disease in Busia and Kakamega counties at the slaughter slab points.

## 5. Conclusions

The free-range pig production system (no fencing and scavenging) and inappropriate use of latrines were the critical poor management practices that propagated and propelled PC infection at the farm level in Busia and Kakamega counties. The meat inspection practice as a factor of biosecurity at slaughter slabs was not adequate in the two counties of Western Kenya. These findings suggested that there is a need for implementation of effective pig biosecurity measures to prevent PC infections and ensure food safety along the pork value chain in Western Kenya. This will require collaboration with policymakers who have in their mandate the reinforcement of the regulations by inspiring farmers through sensitization training and strengthening the meat inspection in Western Kenya.

## Figures and Tables

**Table 1:** Management practices, type of questions, and responses.

Management practices	Type of questions	Responses
Free-range pig keeping	Are your pigs kept outdoors?	Yes/no
Use of outdoor defecation	Are outdoor bushes used for defecation?	Yes/no
Presence of latrine at the household	Does the household own a pit latrine?	Yes/no
Use of pit latrine by the household	Do the household members use the pit latrine?	Yes/no
Sourcing water outside the farm	Whether farmers sourcing water outside the farm or not?	Yes/no
Sourcing feed outside the farm	Whether farmers sourcing feed outside the farm or not?	Yes/no
Routine deworming	Are often your pigs dewormed?	Yes/no
Routine vaccination	Whether farmer vaccinated pigs or not?	Yes/no
Presence of a fenced farm	Whether farm had a fenced pigpen or not?	Yes/no
Meat inspection	Whether meat was inspected at the slaughter slabs in Busia/Kakamega or not?	Yes/no

**Table 2:** Farmers' demographic characteristics (*n* = 162).

Variables	Statement	Frequency	Percent
Age (years)	11–20	41	25.3
	21–30	61	37.7
	31–40	43	25.5
	41–50	17	10.5
Gender	Male	76	46.9
	Female	86	53.1
Education level	None	67	41.4
	Primary	41	25.3
	Secondary	46	28.4
	College/university	8	4.9
Farmer occupation	Farming	153	94.4
	Public employee	4	2.5
	Private employee	5	3.1
Farmers' pig production experience (years)	10-Jan	125	77.2
	20-Nov	31	19.1
	21–30	4	2.5
	31–40	1	0.6
	41–50	1	0.6

**Table 3:** Management practices implemented at the farm level within Busia and Kakamega counties (*n* = 162).

Management practices	Practice	Count	Percent	OR	*p* value
Free-range pig keeping	Frequently	112	69.1	2.24	<0.0001
	Not frequently	50	30.9		
Use of outdoor defecation by humans	Frequently	54	33.3	0.50	<0.0001
	Not frequently	108	66.7		
Presence of latrine at the household	Frequently	142	87.6	7.10	<0.0001
	Not frequently	20	12.4		
Use of latrine by the household	Frequently	118	72.8	2.68	<0.0001
	Not frequently	44	27.2		
Sourcing water outside the farm	Frequently	13	8	0.09	<0.0001
	Not frequently	149	92		
Sourcing feed outside the farm	Frequently	21	13	0.15	<0.0001
	Not frequently	141	87		
Routine deworming	Frequently	48	29.6	0.42	<0.0001
	Not frequently	114	70.4		
Routine vaccination	Frequently	49	30.3	0.43	<0.0001
	Not frequently	113	69.7		
Presence of a fenced farm	Frequently	36	22.2	0.29	<0.0001
	Not frequently	126	77.8		

**Table 4:** Assessment of meat inspection implementation by respondents in the two counties.

Respondents	Meat inspection	Frequency	Percent	OR	*p* value
Butcher owners	Frequently	20	76.9	3.3	0.006
	Not frequently	6	23.1		
Workers	Frequently	16	61.5	1.6	0.2393
	Not frequently	10	38.5		

## Data Availability

The data used to support the findings of this study are available from the corresponding author upon request.
